# Autoantibody of NRIP, a novel AChR‐interacting protein, plays a detrimental role in myasthenia gravis

**DOI:** 10.1002/jcsm.12697

**Published:** 2021-03-26

**Authors:** Li‐Kai Tsai, I‐Hsin Chen, Chi‐Chao Chao, Hsueh‐Wen Hsueh, Hsin‐Hsiung Chen, Yun‐Hsin Huang, Rong‐Wei Weng, Tzu‐Yun Lai, Yi‐Chieh Tsai, Yeou‐Ping Tsao, Show‐Li Chen

**Affiliations:** ^1^ Department of Neurology National Taiwan University Hospital Taipei Taiwan; ^2^ Department of Neurology National Taiwan University Hospital, Hsinchu Branch Hsinchu Taiwan; ^3^ Graduate Institute of Microbiology, College of Medicine National Taiwan University Taipei Taiwan; ^4^ Department of Ophthalmology Mackay Memorial Hospital Taipei Taiwan

**Keywords:** Autoantibody; Acetylcholine receptor; Myasthenia gravis; Neuromuscular junction; Nuclear receptor interaction protein

## Abstract

**Background:**

Nuclear receptor interaction protein (NRIP) co‐localizes with acetylcholine receptor (AChR) at the neuromuscular junction (NMJ), and NRIP deficiency causes aberrant NMJ architecture. However, the normal physiological and pathophysiological roles of NRIP in NMJ are still unclear.

**Methods:**

We investigated the co‐localization and interaction of NRIP with AChR‐associated proteins using immunofluorescence and immunoprecipitation assay, respectively. The binding affinity of AChR‐associated proteins was analysed in muscle‐restricted NRIP knockout mice and NRIP knockout muscle cells (C2C12). We further collected the sera from 43 patients with myasthenia gravis (MG), an NMJ disorder. The existence and features of anti‐NRIP autoantibody in sera were studied using Western blot and epitope mapping.

**Results:**

NRIP co‐localized with AChR, rapsyn and α‐actinin 2 (ACTN2) in gastrocnemius muscles of mice; and α‐bungarotoxin (BTX) pull‐down assay revealed NRIP with rapsyn and ACTN2 in complexes from muscle tissues and cells. NRIP directly binds with α subunit of AChR (AChRα) *in vitro* and *in vivo* to affect the binding affinity of AChR with rapsyn and rapsyn with ACTN2. In 43 patients with MG (age, 58.4 ± 14.5 years; female, 55.8%), we detected six of them (14.0%) having anti‐NRIP autoantibody. The presence of anti‐NRIP autoantibody correlated with a more severe type of MG when AChR autoantibody existed (*P* = 0.011). The higher the titre of anti‐NRIP autoantibody, the more severe MG severity (*P* = 0.032). The main immunogenic region is likely on the IQ motif of NRIP. We also showed the IgG subclass of anti‐NRIP autoantibody mainly to be IgG1.

**Conclusions:**

NRIP is a novel AChRα binding protein and involves structural NMJ formation, which acts as a scaffold to stabilize AChR–rapsyn–ACTN2 complexes. Anti‐NRIP autoantibody is a novel autoantibody in MG and plays a detrimental role in MG with the coexistence of anti‐AChR autoantibody.

## Introduction

The nuclear receptor interaction protein (NRIP, also named DCAF6 and IQWD1) consists of 860 amino acids with seven WD40 repeats and one IQ motif.^1^, ^2^ NRIP regulates muscle contraction via interaction with calmodulin (CaM) through the IQ motif to activate calcineurin and CaM kinase II signalling.^3^, ^4^ Muscular NRIP is also a trophic factor retrogradely supporting spinal motor neuron via the stabilization of neuromuscular junction (NMJ) by myogenin expression.^5^ In addition, NRIP contributes to muscle regeneration upon muscle injury during myogenesis and myotube formation.^4^ Subsequently, both global NRIP knockout and muscle‐restricted NRIP knockout mice showed impaired motor behavioural performances.^4^, ^5^ In patients with limb‐girdle muscular dystrophy, NRIP expression is reduced in dystrophic muscles.^6^ Therefore, NRIP is a muscular protein playing an important role in maintaining normal neuromuscular integrity and function.

Myasthenia gravis (MG) is an autoimmune‐related neuromuscular disorder resulting from an attack of NMJ by autoantibodies. The NMJ injury leads to muscle weakness, difficulty in ambulation, respiratory failure or even death.^7^ A diagnosis of MG is essentially based on clinical presentations such as ptosis, diplopia, dysarthria and weakness of the four limbs and laboratory tests, including the presence of MG autoantibodies and electrophysiological studies.^8^ The treatment for MG targets the immune system (immunosuppressant or immunomodulation) and NMJ (cholinesterase inhibitors).^9^ However, some patients with MG show low therapeutic responses, and discovery of more effective and specific treatment is needed.^10^

We have previously demonstrated that muscular NRIP co‐localized with a major NMJ protein, acetylcholine receptor (AChR).^5^ Deprivation of NRIP in skeletal muscles reduced the NMJ area with denervation of AChR.^5^ NRIP probably plays a specific physiological role on NMJ. Besides, the anti‐AChR antibody is the most common autoantibody (75%–80%) in MG, followed by anti‐muscle‐specific kinase (MuSK) and anti‐low‐density lipoprotein receptor‐related protein 4 (LRP4), respectively. However, 10% of MG patients are seronegative,^10^ which indicates the existence of certain unknown NMJ components with unknown autoantibodies for MG. We hypothesized that NRIP is an essential component in NMJ and anti‐NRIP autoantibody contributes to the pathophysiology of MG. Using NRIP knockout cultured cells and muscular restricted NRIP knockout mice, we demonstrated that NRIP is a novel AChR‐interacting protein and NMJ component protein, which acts as a scaffold to stabilize AChR–rapsyn–α‐actinin 2 (ACTN2) complexes. By analysing sera from patients with MG, we showed that anti‐NRIP autoantibody is a novel autoantibody and plays a deleterious role in MG.

## Materials and methods

### Immunofluorescence assay

Gastrocnemius muscles from C57BL/6J mice were collected for the whole mounting stain.^5^ Briefly, the muscle was fixed with 2% paraformaldehyde for 2 h at room temperature, embedded in optimum cutting temperature (OCT) compound, and sectioned to 20 μm thickness. Sections were incubated with anti‐NRIP (GeneTex, Irvine, CA, USA; 1:200), anti‐rapsyn (Abcam, Cambridge, MA, USA; 1:100) and anti‐ACTN2 (Abcam; 1:500) primary antibodies overnight at 4°C and then incubated with a mixture containing 488‐conjugated donkey anti‐rabbit secondary antibody and Alexa‐594‐conjugated α‐bungarotoxin (α‐BTX) and mounted in DAPI Fluoromount‐G (SouthernBiotech, Birmingham, AL, USA). The fluorescent images were visualized by Leica TSC SP5 confocal microscope (Leica Microsystems, Wetzlar, Germany).

### Cell culture

HEK293T and C2C12 cells were cultured in DMEM (Dulbecco's modified Eagle's medium) with 10% fetal bovine serum, 1% penicillin–streptomycin (Thermo Fisher, Carlsbad CA) and 1% L‐glutamine and incubated at 37°C, 5% CO_2_. Cells were passaged every 2 days. For the C2C12 differentiation, cells were washed twice with PBS and applied to the differentiation medium (DMEM with 2% horse serum and 1% penicillin–streptomycin and 1% L‐glutamine). Cells were incubated at 37°C, 5% CO_2_, and differentiated for 5 days.

### HEK293T cell transfection

HEK293T cells were seeded in a 10 cm dish 1 day before transfection. When 293T cells were grown to 70%–80% confluence, the target plasmids were transfected by jetPRIME (Polyplus‐transfection, France) as manufacturer's recommendation (the ratio between DNA and jetPRIME transfection reagent is 1:2 or 1:3; total DNA is 10–15 μg/plate).

### Immunoprecipitation

Equal number of protein lysates was incubated with immunoprecipitation buffer (radioimmunoprecipitation assay buffer without 1% NP‐40, 1% protease inhibitor and 1% phosphatase inhibitor cocktails) and primary antibodies at 4°C overnight with constant mixing. 50 μL protein G Sepharose beads (GE Healthcare Life Sciences, Chicago, IL, USA) were added to the lysates and incubated at 4°C for 2 h and centrifuged at 8000 rpm, 1 min, 4°C, and washed with immunoprecipitation buffer twice. The target proteins were eluted by 5× sample dye and performed Western blot subsequently.

### Recombinant proteins from bacteria

The GST and GST‐AChR fusion proteins were expressed in *Escherichia coli* Rosetta (Merck, Darmstadt, Germany) cells at 16°C. According to the manufacturer's procedures, further purification was performed using glutathione Sepharose beads (GE Healthcare Life Sciences). His‐tagged NRIP protein was purified by Ni–NTA agarose beads (Qiagen, Hilden, Germany). The eluted proteins were analysed by sodium dodecyl sulfate–polyacrylamide gel electrophoresis and Coomassie blue staining.

### Muscle‐restricted NRIP knockout mice

The *NRIP‐LoxP* heterozygous (*NRIP* ^*flox/+*^) mice have been generated, and muscle *creatine kinase (MCK)‐Cre* heterozygous (*Cre* ^*+*^) mice have been obtained as previously described.^5^ We crossed the *NRIP* ^*flox/+*^ and *Cre* ^*+*^ mice to get *NRIP* ^*flox/+*^ *Cre* ^*+*^ mice. We got *NRIP* ^*flox/flox*^ *Cre* ^*+*^ (muscle‐restricted NRIP conditional knockout) and *NRIP* ^*flox/flox*^ *Cre* ^*−*^ mice (wild type) via crossing the *NRIP* ^*flox/+*^ *Cre* ^*+*^ mice to each other.

### Protein extraction and Western blot

HEK293T cells were harvested, and total protein was extracted by treating lysates with RIPA lysis buffer (137 mM NaCl, 20 mM Tris–HCl pH 8.0, 2 mM EDTA, 1% NP‐40), 1% protease inhibitor (Roche, Grenzacherstrasse, Switzerland) and phosphatase inhibitor cocktails (Sigma, Louis, MO, USA) on ice for 20 min. As for C2C12 cells, RIPA lysis buffer was added to 0.1% of sodium dodecyl sulfate additionally. Protein lysates were clarified by centrifugation at 13 200 rpm for 20 min at 4°C, and the supernatant was collected and stored at −80°C. Proteins were separated on sodium dodecyl sulfate–polyacrylamide gel electrophoresis and transferred to polyvinylidene fluoride membranes (Millipore, Temecula, CA, USA) for 2 h. Then, the membrane was blocked in 5% BSA for 1 h at room temperature. The membranes were then incubated with primary antibodies, including anti‐NRIP (Novus, Littleton, CO, USA; 1:2000), anti‐GFP (Abcam; 1:10000), anti‐GAPDH (AbFrontier, Seoul, Korea, 1:10000), anti‐α‐subunit of AChR (AChR‐α; Sigma, 1:1000), anti‐flag (Sigma; 1:2000), anti‐GST (Santa Cruz, Santa Cruz, CA, USA; 1:10000), anti‐His (Proteintech, Rosemont, IL, USA, 1:10000) or sera from MG patients and healthy controls (1:1000), diluted in the blocking buffer and incubated overnight at 4°C. On the next day, membranes were incubated with corresponding HRP‐conjugated secondary antibody (1:10000), anti‐human IgG (Abcam; 1:10000), anti‐human IgG1–3 (Thermo Fisher, Waltham, MA, USA; 1:500) or anti‐human IgG4 (Thermo Fisher; 1:1000) for 1 h at room temperature. The target protein expression was detected using an ECL Western blot detection system (GE Healthcare Life Sciences).

### Patients

Forty‐three MG patients were included in this study. The diagnosis of MG was based on typical clinical manifestations such as ocular symptoms (diplopia and ptosis), dysarthria/dysphagia or proximal‐predominant weakness with abnormal results in electrophysiological tests (repetitive stimulation test or single‐fibre electromyography) or high serum titre of anti‐AChR autoantibody.^8^ The patients were classified according to the Myasthenia Gravis Foundation of America Clinical Classification (MGFACC).^11^ The Class I MG was defined by patients only having ocular symptoms such as diplopia or ptosis, and all other muscle strength is normal. The Class II, III and IV MG were defined by patients having mild, moderate and severe weakness affecting other than ocular muscles, respectively. Class V MG was defined by patients receiving intubation with or without mechanical ventilation, except when employed during routine post‐operative management. The MG was also classified into the ocular and general type or presence of thymoma or not. Respiratory support was defined using bilevel positive airway pressure (BiPAP) or requiring intubation. The stationary MG was defined as no add‐on or titration of medication specifically for MG in the past 1 year. Patients were also classified according to whether they were ever hospitalized for MG except for hospitalization for MG diagnosis or thymectomy. Neurologists who took care of MG patients and collected blood samples were blinded to the test results for anti‐NRIP autoantibody.

### Statistical analysis

All the data analyses and graphs were performed using Microsoft Excel (Microsoft Corp.) and GraphPad Prism (GraphPad Software). Data were presented as mean ± standard deviation, except for the protein binding affinity experiment (mean ± standard error). Results were analysed by Student's *t*‐test or Mann–Whitney *U* test for continuous variables and Fisher's exact test for categorical variables. The Pearson correlation coefficient *r* analysed the correlation. *P*‐value of less than 0.05 was considered statistically significant.

### Study approval

All animal experiments were reviewed and approved by the Institutional Animal Care and Use Committee (IACUC) at the College of Medicine, National Taiwan University. All experimental mice were housed in the animal centre under a 12‐h light–dark cycle with free access to food and water. The human study was conducted with the approval of and under the guidelines of the institutional review board (201811017RIND) in National Taiwan University Hospital and the written informed consent was obtained from all participants or their family members.

## Results

### NRIP is a structural component of NMJ complexes

**Figure 1:**
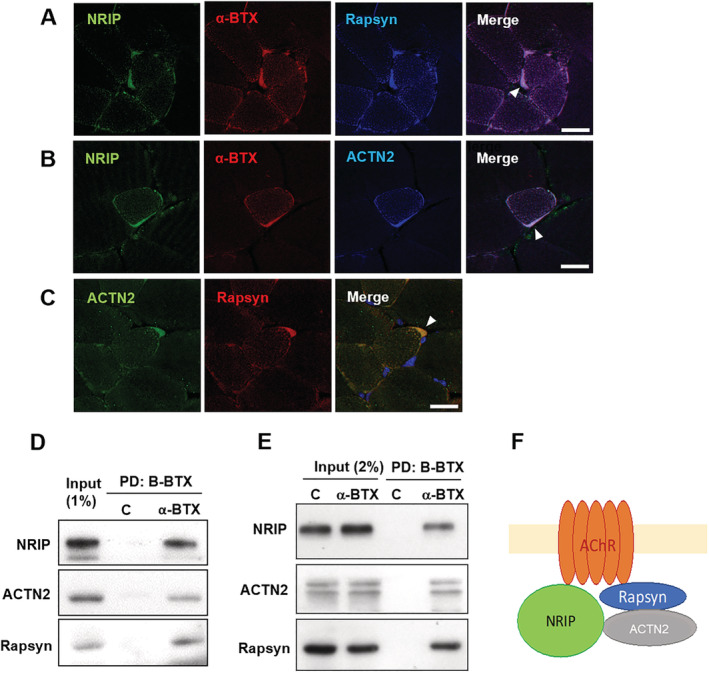
NRIP is a structural component of neuromuscular junction (NMJ) complexes. *(A)* Immunofluorescence assay (IFA) with anti‐NRIP antibody (green), α‐bungarotoxin [α‐BTX; red, for acetylcholine receptor (AChR)] and anti‐rapsyn antibody (blue) in paraffin sections of gastrocnemius muscles from 16‐week‐old wild‐type mice. NRIP co‐localizes with AChR and rapsyn at NMJ. Arrowhead indicates co‐localization. Scale bar, 20 μm. *(B)* IFA with anti‐NRIP (green), α‐BTX (red) and anti‐α‐actinin (ACTN2; blue) antibodies show co‐localization of NRIP with AChR and ACTN2. *(C)* IFA with anti‐ACTN2 (green) and anti‐rapsyn (red) antibodies and DAPI (blue) reveals co‐localization of ACTN2 and rapsyn. *(D)* The protein lysates of gastrocnemius muscles from wild‐type mice were incubated with biotin‐labelled BTX (B‐BTX), which was pulled down (PD) with streptavidin‐coupled agarose beads; control (c) was streptavidin‐coupled agarose beads only. The pull‐down BTX component contains NRIP, rapsyn and ACTN2, indicating that NRIP is associated with AChR–rapsyn–ACTN2 complexes. *(E)* The B‐BTX pull‐down assay in the C2C12 cells shows the association of AChR with NRIP, rapsyn and ACTN2. *(F)* The proposed model of the association of NRIP with AChR, rapsyn and ACTN2.

AChR is a primary structural component of post‐synaptic NMJ; AChR, rapsyn and ACTN2 form a functional complex.^12^ To examine whether NRIP localizes at NMJ with AChR–rapsyn–ACTN2 complex in muscle, immunofluorescence assay was performed in paraffin sections of gastrocnemius muscle from 16‐week‐old wild‐type mice. Using α‐BTX (for AChR), anti‐NRIP, anti‐rapsyn and anti‐ACTN2 antibodies, we demonstrated that NRIP is well co‐localized with AChR, rapsyn and ACTN2 (*Figure* 1A–C).

To confirm the association between NRIP and AChR in the muscle tissue and cultured myocytes, we performed the biotin‐labelled BTX pull‐down assay. The protein lysates from gastrocnemius muscles of wild‐type mice were incubated with biotin‐labelled BTX, pulled down by streptavidin‐coupled agarose beads. The BTX pull‐down component contains NRIP, rapsyn and ACTN2, indicating that NRIP is associated with the AChR–rapsyn–ACTN2 complex (*Figure* 1D). Besides, in a post‐differentiative cultured myocyte cell line, C2C12, the BTX pull‐down component also contains NRIP, rapsyn and ACTN2 (*Figure* 1E). Therefore, NRIP is a novel structural component of NMJ complexes associated with AChR, rapsyn and ACTN2 (*Figure* 1F).

### NRIP is a novel AChRα binding protein

**Figure 2:**
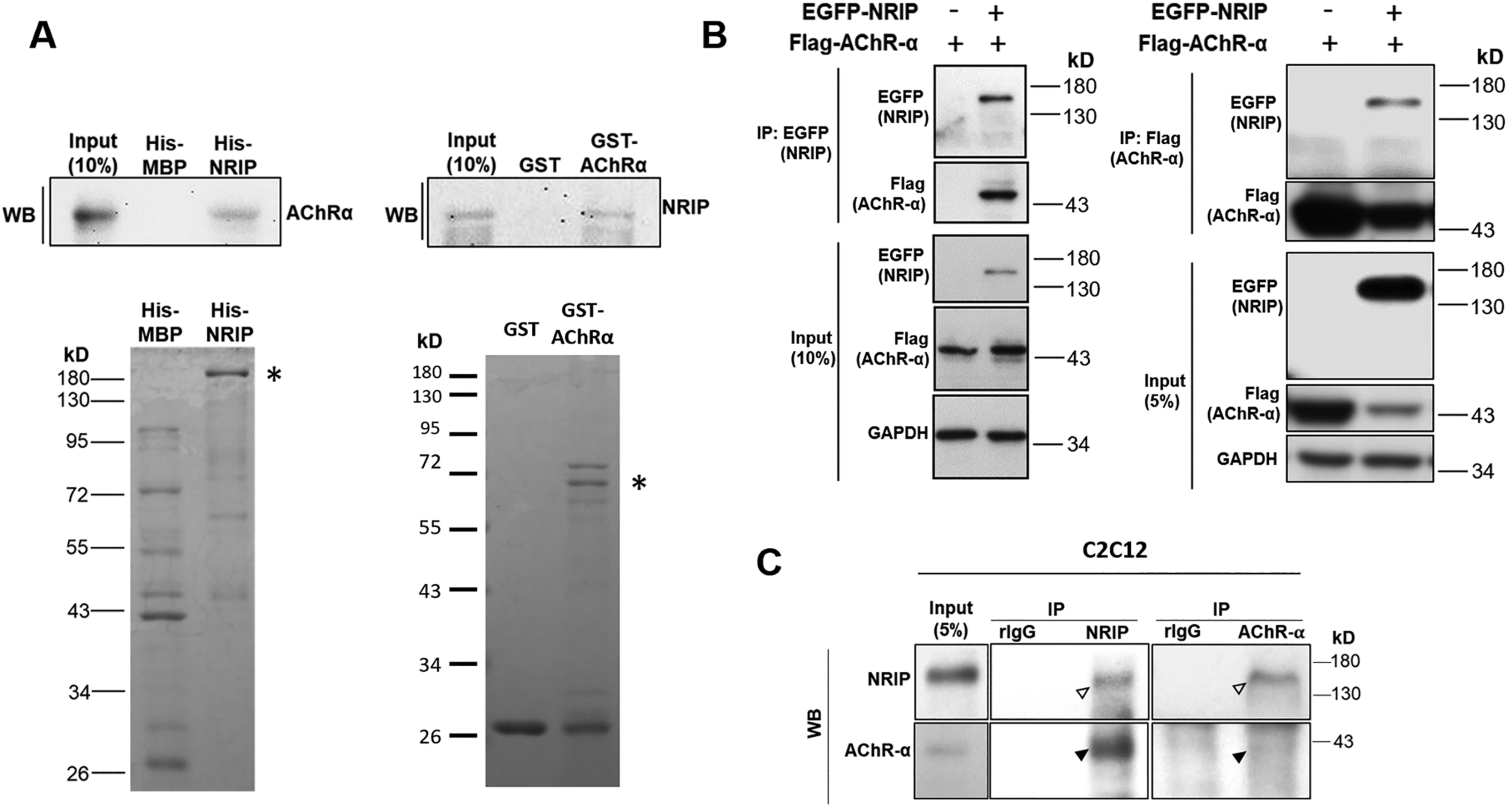
NRIP is a novel AChRα binding protein. *(A) In vitro* direct binding between NRIP and AChRα. His‐MBP‐NRIP and GST‐AChRα proteins produced from bacteria were separately purified by Ni–NTA and GST beads, respectively, and examined by Coomassie blue staining (lower panels), respectively. The asterisks indicate His‐MBP‐NRIP or GST‐AChRα. Each purified protein was incubated together for direct interaction, followed by western blot analysis with antibodies specific against His (left, upper) or GST (right, upper), respectively. *(B)* Reciprocally binding of NRIP and AChRα in HEK293T cells. HEK293T cells were transfected with EGFP‐NRIP and Flag‐AChRα. The immunoprecipitates with anti‐EGFP (NRIP) or anti‐Flag (AChRα) were analysed by Western blot, showing the appearance of AChRα and NRIP, respectively. *(C)* Endogenous interaction of NRIP and AChRα in C2C12 cells. Protein lysates of C2C12 cells, which had differentiated for 5 days, were subjected to immunoprecipitation with anti‐NRIP, anti‐AChRα or normal rabbit IgG (rIgG) separately; the bound proteins were analysed using Western blot. The hollow arrowhead indicates NRIP, and the solid arrowhead indicates AChRα.

To confirm the direct binding between AChRα and NRIP *in vitro*, we first produced recombinant proteins His‐MBP‐NRIP and GST‐AChRα from bacteria with purification using Ni–NTA and GST beads, respectively. Two recombinant proteins were then incubated together for direct interaction, followed by immunoprecipitation and Western blotting using antibodies specifically against His (NRIP) or GST (AChRα). The results showed a reciprocal binding of NRIP and AChRα *in vitro* (*Figure* 2A). To test the binding of NRIP and AChRα in cultured cells, HEK293T cells were transfected with EGFP‐NRIP and Flag‐AChRα. The immunoprecipitates with anti‐EGFP (NRIP) or anti‐Flag (AChRα) were analysed by Western blot, showing the appearance of AChRα and NRIP, respectively, indicating a reciprocal binding of NRIP and AChRα in the cellular system (*Figure* 2B). We further investigated the endogenous binding of NRIP and AChRα in the cultured C2C12 cells. Protein lysates of C2C12 cells, which had differentiated for 5 days, were subjected to immunoprecipitation with anti‐NRIP or anti‐AChRα antibody, respectively. The following Western blot showed a reciprocal binding of NRIP and AChRα again, implying the binding of endogenous NRIP and AChRα in cultured myocytes (*Figure* 2C). In sum, NRIP is a novel AChR binding protein.

### NRIP stabilizes protein–protein interaction of AChR–rapsyn–ACTN2 complex

**Figure 3:**
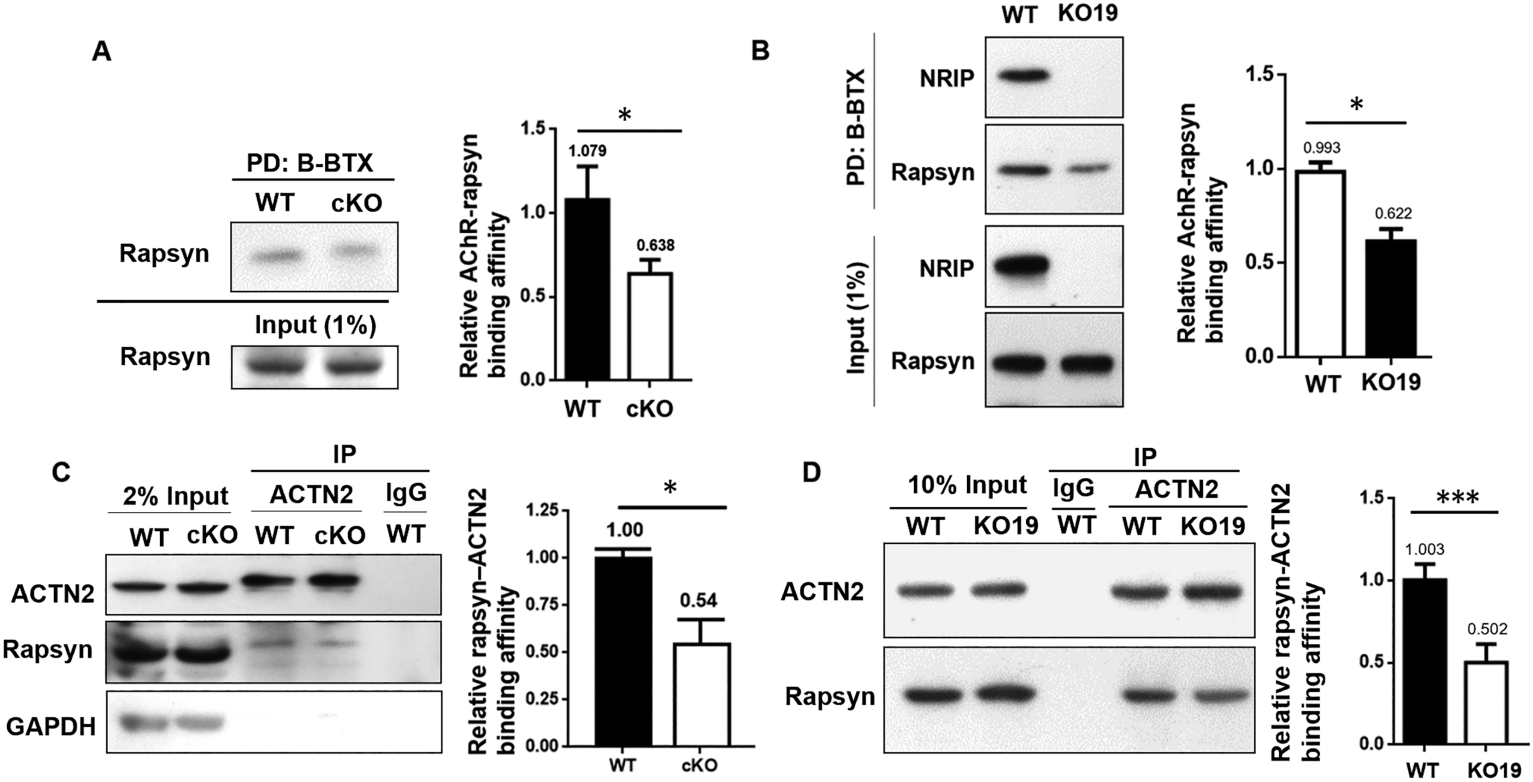
NRIP stabilizes the protein–protein interaction of AChR–rapsyn–ACTN2 complexes. *(A)* Protein lysates were extracted from gastrocnemius muscles of wild‐type (WT) or muscle‐restricted NRIP conditional knockout (cKO) mice. The B‐BTX pull‐down assay followed by Western blot for rapsyn showed a lower binding affinity between AChR and rapsyn in cKO than WT mice. *(B)* Protein lysates were extracted from post‐differentiative C2C12 cells without (WT) or with (KO19) NRIP knockout. The B‐BTX pull‐down assay showed a lower binding affinity between AChR and rapsyn in KO19 than WT C2C12 cells. *(C)* Although the expression of rapsyn and ACTN2 was comparable in gastrocnemius muscles of WT and NRIP cKO mice, the binding affinity between rapsyn and ACTN2 in NRIP cKO mice was reduced than that in mice. *(D**)*** The binding affinity between rapsyn and ACTN2 was lower in KO19 than WT C2C12 cells. The bar‐grams show quantification of relative binding affinity from three separate experiments. The result from one of WT sample was set as 1. Data are mean ± standard error by Student's *t*‐test. ^*^ *P* < 0.05 and ^***^ *P* < 0.001.

While NRIP is a structural component of the NMJ complex with direct interaction with AChR, we hypothesized that NRIP could stabilize the protein–protein interaction of the AChR–rapsyn–ACTN2 complex. We compared the binding affinity of AChR and rapsyn in gastrocnemius muscles of wild‐type and muscle‐restricted NRIP conditional knockout mice using the B‐BTX pull‐down assay. The following Western blot for rapsyn showed lower binding affinity between AChR and rapsyn in NRIP knockout than wild‐type mice (*Figure* 3A; 0.638 vs. 1.079). Previously, we generated NRIP knockout C2C12 cells (using CRISPR Cas9) and named KO19. The B‐BTX pull‐down assay showed a lower binding affinity between AChR and rapsyn in KO19 than wild‐type C2C12 cells (*Figure* 3B; 0.622 vs. 0.993).

Similarly, we used muscle‐restricted NRIP knockout mice and NRIP knockout C2C12 cells to test the binding affinity between rapsyn and ACTN2. Although the expression of rapsyn and ACTN2 was comparable in gastrocnemius muscles of wild‐type and NRIP knockout mice, the binding affinity between rapsyn and ACTN2 in NRIP knockout mice was reduced than that in wild‐type mice (*Figure* 3C; 0.54 vs. 1.00). The binding affinity between rapsyn and ACTN2 is also lower in KO19 than wild‐type C2C12 cells (*Figure* 3D; 0.502 vs. 1.003). Taken together, NRIP strengthens binding affinity between AChR and rapsyn as well as between rapsyn and ACTN2 in both mouse muscles and C2C12 cells and acts as a scaffold protein stabilizing the AChR–rapsyn–ACTN2 complex.

### Anti‐NRIP autoantibodies in patients with MG

**Figure 4:**
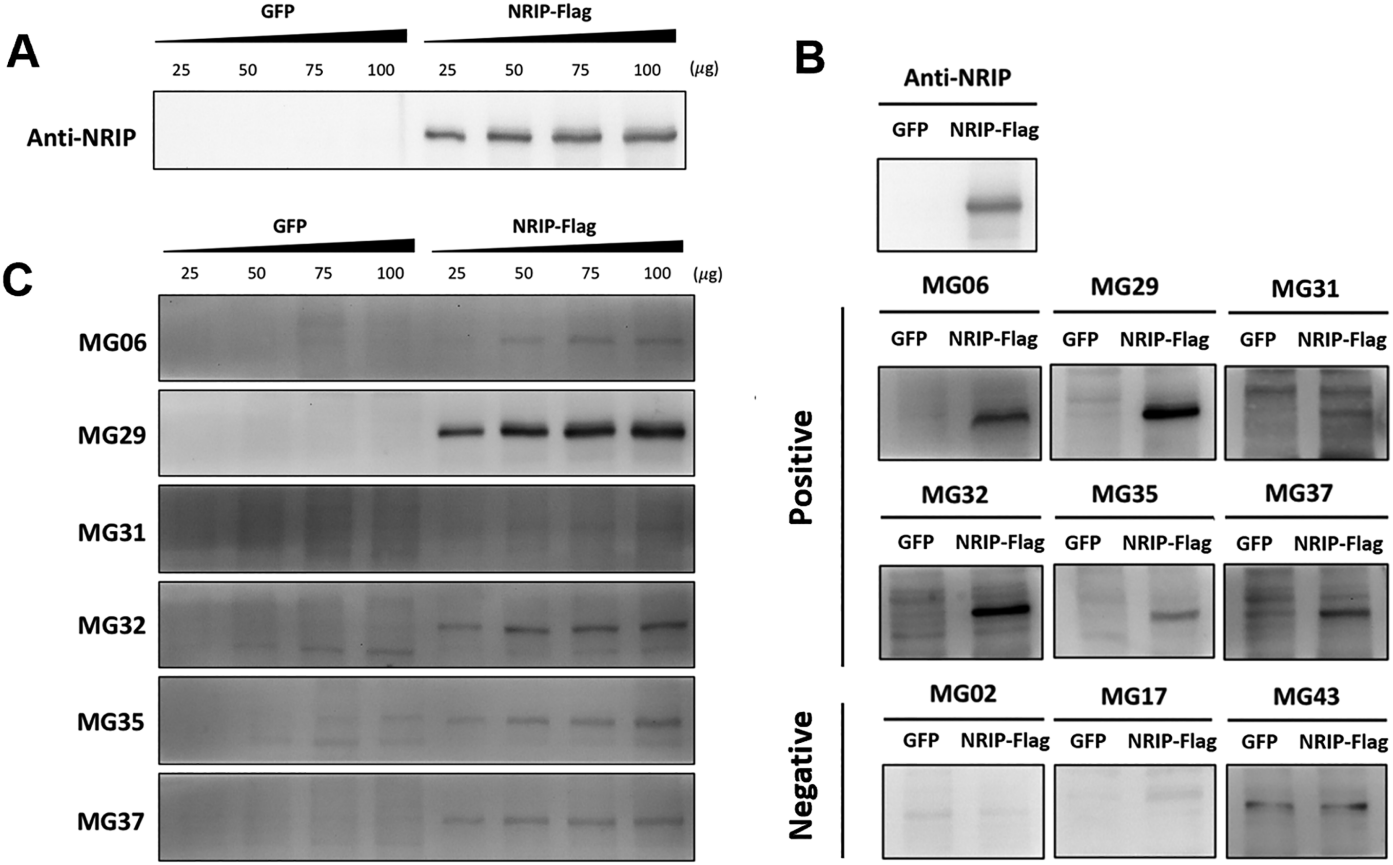
Detection of anti‐NRIP autoantibody in patients with myasthenia gravis (MG). *(A)* Using different doses of NRIP‐Flag for Western blot shows that the protein quantity of 50 μg was enough to be used for autoantibody detection. *(B)* The transfected protein lysates with GFP or NRIP‐Flag were used as antigens in Western blot, and GFP was used as the negative control. The sera of 43 patients with MG, which were diluted at a ratio of 1:1000, were used as the primary antibodies. Sera showing positive signals with NRIP‐flag but negative with GFP are anti‐NRIP seropositive. *(C)* In six anti‐NRIP seropositive patients, the sera reacted to NRIP‐Flag in a dose‐dependent manner along with the increase of NRIP‐Flag quantity.

To investigate whether the anti‐NRIP autoantibody exists in sera of MG patients, we first produced NRIP protein from HEK293T cells using various methods through transfection or viral vector infection. After 48 h, the NRIP expression was analysed using Western blot. Among different methods, NRIP‐Flag produced by pAAV‐NRIP‐Flag plasmid transfection (*Figure* *S1*, lane 6) had the best expression efficacy. Therefore, NRIP‐Flag produced by this way was served as the antigen, and the GFP protein expressed via pAAV‐GFP transfection by jetPRIME was served as the negative control in further Western blotting analysis. We used 50 μg of NRIP‐Flag to be a standard amount after testing different doses of NRIP‐Flag to determine the optimal quantity (*Figure* 4A). Subsequently, the protein lysates from pAAV‐NRIP‐Flag transfected HEK293T cells were used as antigens, and the sera from patients with MG were used as primary antibodies in Western blot to detect the anti‐NRIP autoantibody.

In 43 patients with MG (age, 58.4 ± 14.5 years; man, 19), 14 (32.6%) had ocular MG, and six (14.0%) ever required respiratory support. Thirty‐three patients (76.7%) were seropositive for the anti‐AChR antibody. Using Western blot, we found that NRIP autoantibodies were detectable in sera from six patients with MG (*Figures* 4B and *S2*). To confirm the presence of anti‐NRIP autoantibodies, different doses of NRIP‐flag were tested in western blot. While all sera from 6 anti‐NRIP seropositive patients did not react to GFP, they reacted to NRIP‐Flag in a dose‐dependent manner along with the increase of NRIP‐Flag quantity (*Figure* 4C). Therefore, we confirmed that anti‐NRIP autoantibody exists in a small group of patients with MG (14.0%).

### The existence of anti‐NRIP autoantibody correlates with MG severity

We compared the clinical features of MG patients with (*n* = 6) and without (*n* = 37) anti‐NRIP autoantibody (Table 1). There is no significant difference between groups regarding age, gender, disease duration, the existence of thymoma, ocular form or not, the requirement of respiratory support or hospitalization or the MGFACC. Notably, all six MG patients with anti‐NRIP autoantibody also have anti‐AChR autoantibody. Patients with MG and anti‐NRIP autoantibody showed a trend to have unstable MG course (requiring titration of MG‐specific medication within 1 year) compared with those without anti‐NRIP autoantibodies, although the difference did not reach statistical significance (*P* = 0.077).

**Table 1:** Features of myasthenia gravis according to the presence of anti‐NRIP autoantibody

		Anti‐NRIP (−) *n* = 37	Anti‐NRIP (+) *n* = 6	*P*‐value
Age (years)		58.7 ± 14.8	56.5 ± 15.0	0.66
Onset duration (years)		6.2 ± 8.3	6 ± 4.2	0.341
Man		17 (45.9)	2 (33.3)	0.678
Anti‐AChR antibody		27 (73.0)	6 (100)	0.323
Thymoma		7 (18.9)	2 (33.3)	0.589
Ocular form		14 (37.8)	0 (0)	0.155
MGFACC	I, II	26 (70.3)	2 (33.3)	0.161
	III, IV, V	11 (29.7)	4 (66.7)	
Respiratory support^a^		4 (10.8)	2 (33.3)	0.19
Hospitalization^b^		13 (35.1)	2 (33.3)	1
Titration of medication^c^		10 (27.0)	4 (66.7)	0.077

Values are number (percentage), except for age and duration (mean ± standard deviation).

AChR, acetylcholine receptor; MGFACC, Myasthenia Gravis Foundation of America Clinical Classification; NRIP, nuclear receptor interaction protein.

^a^ Patients ever required respiratory support with a ventilator or biphasic positive airway pressure.

^b^ Hospitalization for acute exacerbation of myasthenia gravis or complication, not including for diagnosis of myasthenia gravis or thymectomy.

^c^ Requiring titration of immunosuppressant or cholinesterase inhibitor for clinical deterioration in 1 year.

**Figure 5:**
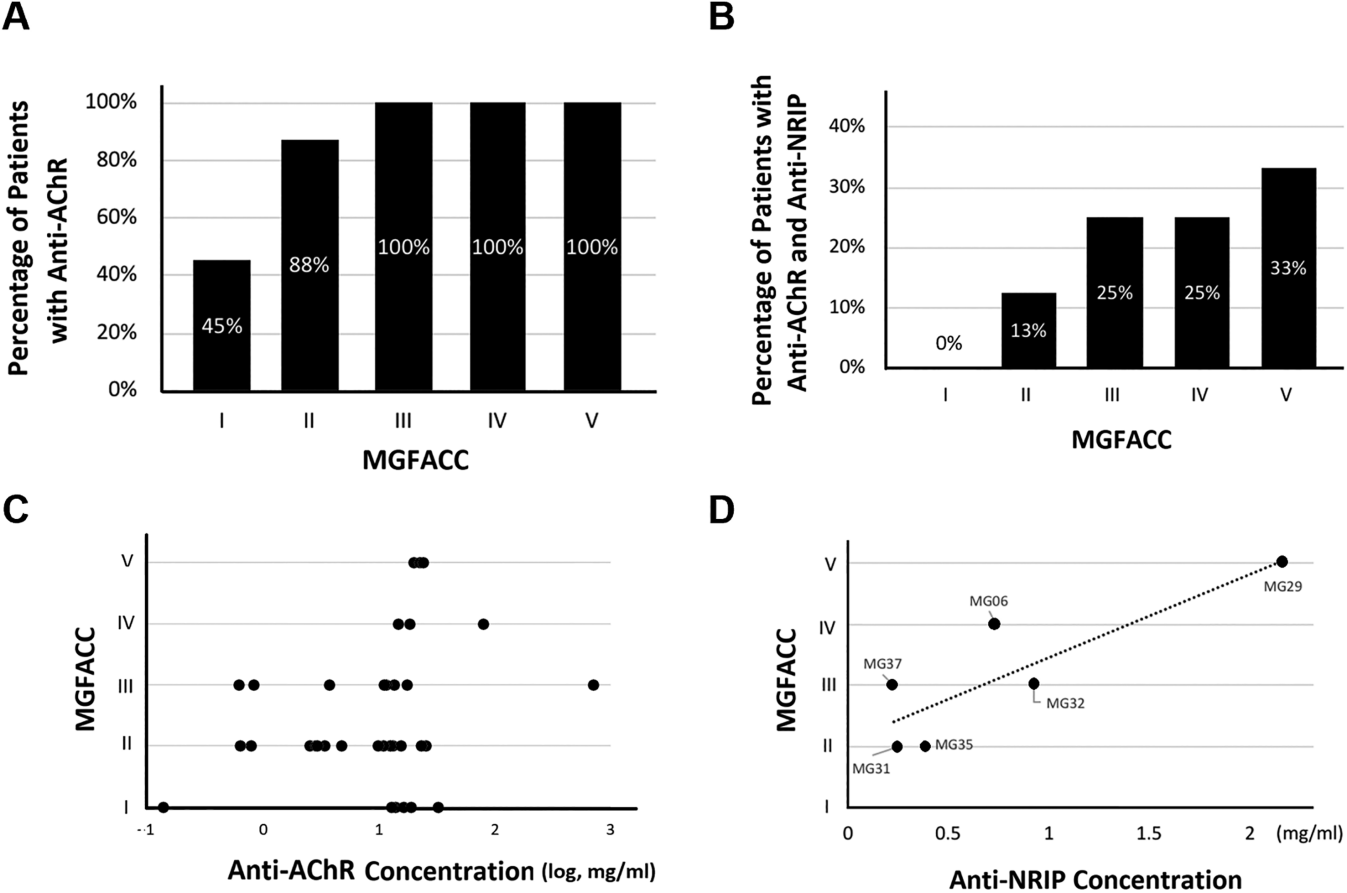
Correlation between anti‐NRIP autoantibodies and MG severity. *(A)* The bar graph represents the percentage of MG patients with anti‐AChR autoantibodies in each MGFACC class. The correlation between MGFACC and the percentage of patients with anti‐AChR autoantibody is calculated by the Pearson correlation coefficient. *r* = 0.810; *P* = 0.097. *(B)* The bar graph represents the percentage of MG patients with both anti‐AChR and anti‐NRIP autoantibodies in each MGFACC class. The severity of MG classified by MGFACC correlates with the percentage of patients having both anti‐AChR and anti‐NRIP autoantibodies. *r* = 0.957; *P* = 0.011. *(C)* The graph represents the titre of anti‐AChR autoantibody and the MGFACC class. *r* = 0.269; *P* = 0.123. *(D)* The titre of anti‐NRIP autoantibody correlates to the MGFACC class in MG patients with seropositive for anti‐NRIP autoantibody. *r* = 0.851; *P* = 0.032.

We further correlated the existence of anti‐AChR or anti‐NRIP autoantibodies with MG severity, stratified by MGFACC class. Although the percentage of MG patients with anti‐AChR autoantibody was not significantly associated with MG severity (*P* = 0.097) (*Figure* 5A), the severity of MG correlated with the percentage of patients having both anti‐AChR and anti‐NRIP autoantibodies (*P* = 0.011) (*Figure* 5B). Besides, we also analysed the relationship between MG severity and the titre of anti‐AChR or anti‐NRIP autoantibodies. Though the titre of anti‐AChR autoantibody was not significantly associated with MG severity (*P* = 0.123) (*Figure* 5C), the severity of MG correlated with serum titre of anti‐NRIP autoantibody (*P* = 0.032) (*Figure* 5D). Taken together, the existence or higher titre of anti‐NRIP autoantibody is associated with more severe disease severity of MG, implying the pathogenicity of anti‐NRIP autoantibody.

### Anti‐NRIP autoantibody against the IQ domain of NRIP

**Figure 6:**
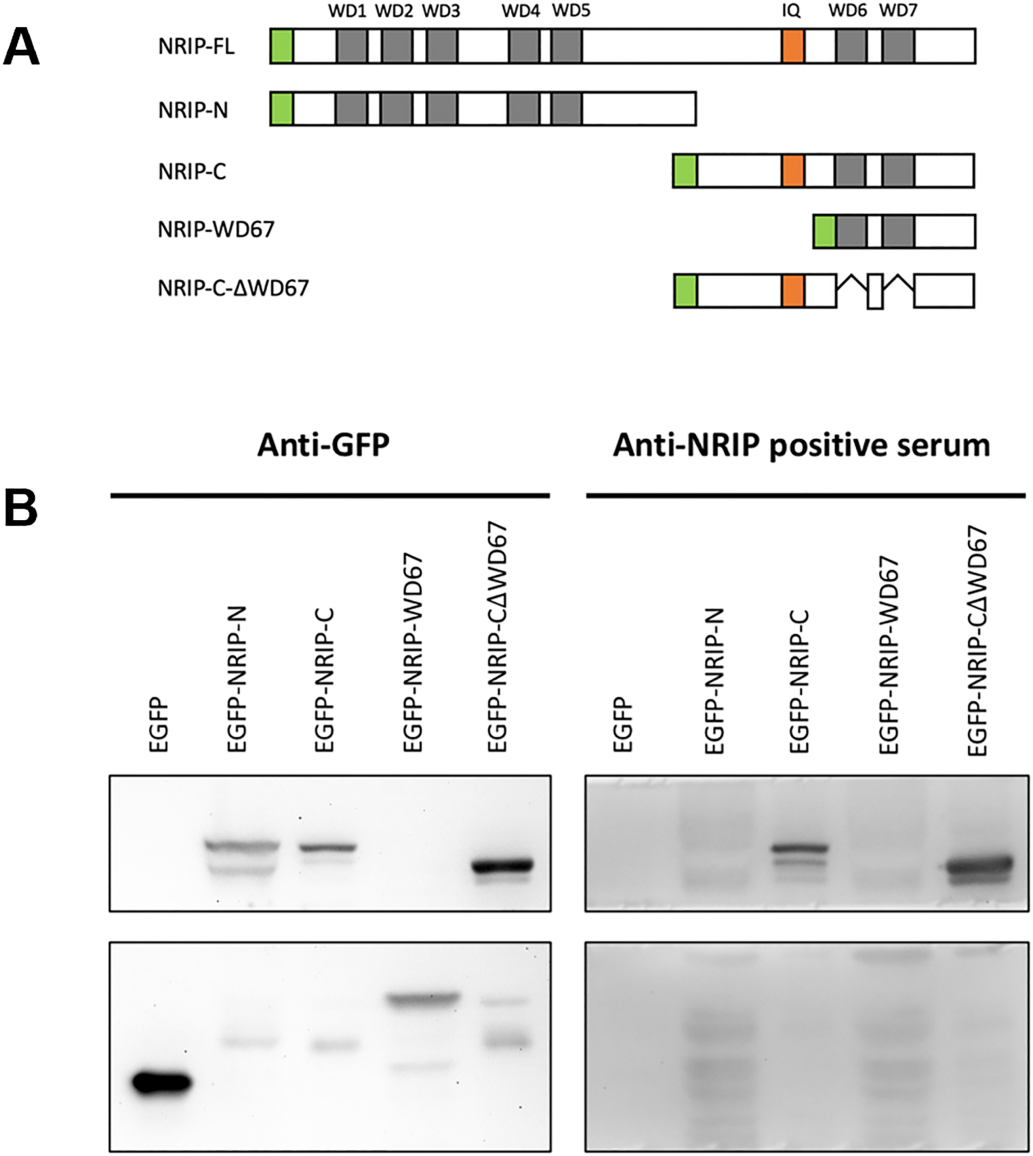
The epitope mapping of anti‐NRIP autoantibody. *(A)* The schematic illustration of NRIP truncated mutants used for epitope mapping. *(B)* The 293T cells were transiently transfected with EGFP or each NRIP truncated mutant for 48 h. Cell lysates were extracted and used for the detection of epitopes using Western blot. The sera of MG patients with anti‐NRIP autoantibodies were used as primary antibodies. Anti‐GFP was used as a positive control. The data shown here are the results of patient MG29. The epitope against NRIP autoantibody is mainly on the *C*‐terminal but excluding WD6 and WD7 domains of NRIP.

For epitope mapping of anti‐NRIP autoantibody, we transfected HEK293T cells with EGFP and EGFP‐NRIP with different NRIP truncated mutants, including full‐length NRIP, *N*‐terminal truncated NRIP, *C*‐terminal truncated NRIP, WD6 and WD7 domains only and *C*‐terminal truncated NRIP with the deletion of WD6 and WD7 domains. The schematic illustration of each NRIP truncated mutant is presented in *Figure* 6A. After 48 h of transfection, cell lysates were extracted and used as epitopes for Western blot. The sera of six MG patients with NRIP autoantibodies were used as primary antibodies. We found that most anti‐NRIP autoantibodies recognized *C*‐terminal truncated NRIP with or without deletion of WD6 and WD7 domains (*Figures* 6B and *S3*). The above results indicate that the epitope against NRIP autoantibody is mainly on the *C*‐terminal but excluding WD6 and WD7 domains of NRIP, likely against the IQ domain of NRIP.

### Most anti‐NRIP autoantibodies belong to the IgG1 subclass

**Figure 7:**
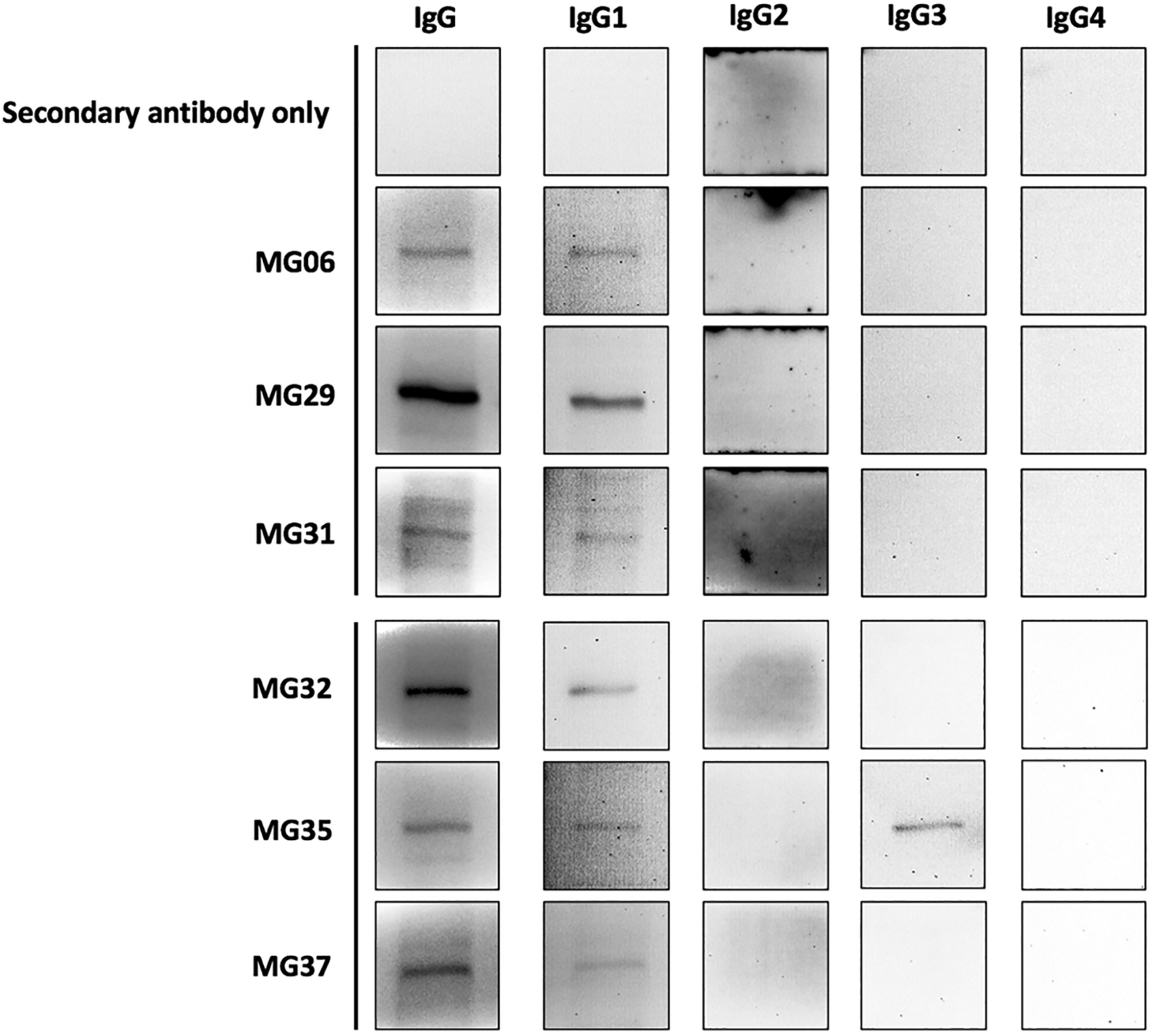
IgG subclass analysis for anti‐NRIP autoantibody. HEK293T cells were transiently transfected with pAAV‐NRIP‐Flag for 48 h. Cell lysates were extracted and used as antigens for the detection of IgG subclass of anti‐NRIP autoantibodies by Western blot. The sera of MG patients were used as primary antibodies. Different secondary antibodies for each human IgG subclass were used. The experiment with secondary antibodies only was served as negative control; the experiment with anti‐human IgG secondary antibody, which can detect all the IgG subclass was served as the positive control. The Western blot shows that all the six anti‐NRIP seropositive patients with MG had anti‐NRIP autoantibody belonging to IgG1, whereas one of them (MG35) also had IgG3 subclass of anti‐NRIP autoantibody.

The immunoglobulin G is composed of four subclasses composed of four subclasses: IgG1, IgG2, IgG3 and IgG4. The different subclass of IgG causes immune responses by a different pathway. To understand the pathogenesis of NRIP autoantibody, we tried to determine the IgG subclass of anti‐NRIP. After transfection of HEK293T cells with pAAV‐NRIP‐Flag, cell lysate was used as antigen in Western blot. The sera of MG patients with anti‐NRIP autoantibody were used as primary antibody and different secondary antibodies for each human IgG subclass. We found that all six anti‐NRIP seropositive patients with MG had anti‐NRIP autoantibody belonging to IgG1, whereas one of them (MG35) also had IgG3 subclass anti‐NRIP autoantibody (*Figure* 7).

### Discussion

Previous studies have shown that muscular NRIP contributes to normal function and integrity of the neuromuscular system.^3^, ^4^, ^5^ In this study, we specifically investigated the normal physiological and pathophysiological roles of NRIP in NMJ. We demonstrated that NRIP co‐localizes with NMJ complex protein and interacts with AChR. NRIP knockout reduced AChR–rapsyn and rapsyn–ACTN2 binding affinity. The above findings indicate that NRIP is a novel NMJ component protein, which acts as a scaffold to stabilize the AChR–rapsyn–ACTN2 complex. In addition, we detected 14% of MG patients having anti‐NRIP autoantibody. The presence and higher titre of anti‐NRIP autoantibody correlated with a more severe type of MG. We also demonstrated that the immunogenic region of NRIP is likely on the IQ motif and anti‐NRIP autoantibody mainly belongs to the IgG1 subclass. Therefore, the anti‐NRIP antibody is a novel autoantibody in MG and plays a detrimental role in MG severity.

NMJ is a chemical synapse between a motor nerve terminal and postsynaptic region on a muscle fibre. When the action potential arrives at the nerve terminal, the presynaptic terminal releases the neurotransmitter acetylcholine, which diffuses across the synaptic cleft and binds to AChR, leading to muscle contraction.^13^ AChR binds to rapsyn, and rapsyn directly interacts with ACTN2 to induce AChR clustering and NMJ stability,^14^, ^15^ which ensure rapid, robust and reliable synaptic transmission.^16^ In this study, we demonstrated that NRIP co‐localizes with AChR, rapsyn and ACTN2 at NMJ of mouse muscle. We further confirmed that exogenous NRIP reciprocally interacts with exogenous α‐subunit of AChR in both test tube and HEK293T cells. Endogenous NRIP also reciprocally interacts with AChR in C2C12 myocytes. Additionally, a previous study showed that NRIP directly interacts with ACTN2 at the Z‐disc of cardiomyocytes.^17^ These results indicate that NRIP is a structural component of AChR–rapsyn–ACTN2 complex at NMJ (*Figure* 1F), having a direct interaction with α‐subunit of AChR and probably ACTN2.

Deprivation of NRIP in skeletal muscles resulted in a small NMJ area with denervation of AChR.^5^ The global or muscle‐restricted NRIP knockout mice displayed impaired motor behaviours.^4^, ^5^ To investigate the function of NRIP at NMJ, we tried to knockout NRIP both *in vivo* and *in vitro* to test the stability of the AChR complex. The binding affinity between AChR and rapsyn as well as rapsyn and ACTN2 reduced in muscle‐restricted NRIP knockout mice and NRIP knockout C2C12 cells. The above findings imply that NRIP acts as a scaffold to stabilize the AChR–rapsyn–ACTN2 complex. Without NRIP to hold the complex together, the efficacy of synaptic transmission and AChR clustering may reduce to cause abnormal NMJ architecture with impaired motor function, as noted in NRIP knockout mice.^5^

MG is resulted from autoantibodies against the postsynaptic membrane of NMJ. The common autoantibodies include anti‐AChR (75%–80%), anti‐MuSK (1%–10%) and anti‐Lrp4 (1%–5%).^10^ In this study, we demonstrated that 14% of patients with MG having anti‐NRIP autoantibody. The dose‐dependent detecting signal using Western blot confirmed the existence of anti‐NRIP autoantibody in sera of this small group of patients with MG (*Figure* 4C). Notably, the presence of anti‐NRIP autoantibody correlated with a more severe type of MG when anti‐AChR autoantibody existed. In addition, the higher the titre of NRIP autoantibody, the more severe the MG severity. Patients with MG and having anti‐NRIP autoantibodies also showed a non‐significant trend to have unstable MG course. Therefore, anti‐NRIP autoantibody did play a detrimental role in MG that patients with MG and having anti‐NRIP autoantibody should be treated cautiously. Future extensive studies are mandatory to confirm the prevalence and clinical features of MG associated with anti‐NRIP autoantibody.

Different autoantibodies of MG attack NMJ via different mechanisms. Anti‐AChR autoantibody, mainly belonging to IgG1 and IgG3 subclasses, usually targets the main immunogenic region of AChR^18^ and causes MG via activation of the classical complement cascade followed by membrane lysis,^19^ triggering the endocytosis of AChR, and prevention of acetylcholine binding to AChR.^20^ However, anti‐MuSK antibody belongs to IgG4 subclass, which blocks MuSK activation, causing the reduced density of AChRs.^21^ On the other hand, the pathogenetic roles of anti‐Lrp4, anti‐agrin and anti‐ColQ autoantibodies are less understood.^8^ In addition to the above autoantibodies targeting extracellular proteins, autoantibodies against intracellular antigens, such as anti‐titin, anti‐ryanodine receptor and anti‐cortactin antibodies, are less likely pathogenetic for MG but markers for MG characteristics, such as disease severity, the presence of thymoma and myopathy.^8^, ^22^ To characterize the features of anti‐NRIP autoantibody, we conducted an epitope mapping study, which showed that the immunogenic region of NRIP is likely on the IQ motif. We also demonstrated that the anti‐NRIP autoantibody mainly belongs to the IgG1 subclass, which theoretically can activate the complement response. Because NRIP did not have a transmembrane domain,^2^, ^3^ NRIP is likely an intracellular protein not exposed to extracellular space for the anti‐NRIP antibody to attack. Considering that all MG patients with seropositive for anti‐NRIP autoantibody also had concomitant anti‐AChR antibody, we hypothesized that anti‐AChR autoantibody first binds AChR with activation of the complement cascade and membrane destruction, leading to the exposure of NRIP. Anti‐NRIP autoantibody subsequently attacks the IQ domain of NRIP and activates more complement responses. Therefore, patients with MG and having both anti‐AChR and anti‐NRIP autoantibodies showed worse outcomes than those without anti‐NRIP autoantibody. In this study, we did not analyse other potential autoantibodies of MG such as anti‐MuSK, anti‐Lrp4, anti‐agrin, anti‐ColQ, anti‐titin, anti‐ryanodine receptor or anti‐cortactin; therefore, the relationship between anti‐NRIP to these autoantibodies is still unclear.

We also collected the sera from 19 healthy controls (mean age, 33.8 ± 12.4 years; man, 10) without ptosis, diplopia or limb weakness. Anti‐NRIP autoantibody can be detected in six (31.6%) (*Figure* *S4*A). Ten of 19 controls received the anti‐NRIP test twice (mean interval of 5.0 ± 0.8 months), and the results of the two analyses were consistent (*Figure* *S4*B). The epitope mapping of anti‐NRIP autoantibodies from six anti‐NRIP seropositive controls showed that most anti‐NRIP autoantibodies targeted the *C*‐terminal of NRIP but without WD6 and WD7 domains (*Figure* *S5*), similar to that in patients with MG. These anti‐NRIP autoantibodies also mainly belonged to the IgG1 subclass (*Figure* *S6*). The titres of anti‐NRIP autoantibody in six patients with MG and six control subjects were similar (0.78 ± 0.67 vs. 0.50 ± 0.47 mg/mL, *P* = 0.522). Taken together, anti‐NRIP autoantibody appears in both patients with MG and control subjects. Without anti‐AChR autoantibody, anti‐NRIP autoantibody probably less likely disturbs NMJ function alone.

In summary, we demonstrated that NRIP is a novel AChR binding protein and NMJ structural component protein, which acts as a scaffold to stabilize the AChR–rapsyn–ACTN2 complex. This finding increases our understanding of the physiology of NMJ complex and expanses our knowledge of NRIP function in the neuromuscular system. Also, we showed that anti‐NRIP autoantibody may be a novel autoantibody in MG, which increases the severity of MG with the coexistence of anti‐AChR antibody.

## Author contribution

Tsai LK and Chen SL conceived and designed the experiments. Chen YS, Chen HH, Huang YH, Weng RW, Lai TZ and Tsai YC performed the experiments. Tsai LK, Chen YS, Weng RW, Tsao YP and Chen SL analysed the data. Tsai LK, Chao CC and Hsueh HW collected the patients. Tsai LK, Chen YS, Chen HH and Chen SL contributed to the writing of the manuscript.

## Conflict of interest

The authors declare no conflict of interest or competing or financial interests.

## Data availability Statement

The authors declare that the data supporting the findings of this study are available within the paper and its Supplementary Information files or available on request from the corresponding author.

## Supporting information


**Figure S1.** NRIP protein was produced from HEK293T cells in different ways, including plasmid transfection with 3xFlag vector by jetPRIME (lane 1); 3xFlag‐NRIPby jetPRIME (lane 2), calcium phosphate with 5 μg 3xFlag‐NRIP (lane 3) or 10 μg 3xFlag‐NRIP (lane 4); with pAAV‐GFP plasmid DNA (lane 5) and pAAV‐NRIP‐Flag plasmid DNA (lane 6) by jetPRIME; and infection by AAV virus encoding NRIP‐Flag (lane 7) and plasmid DNA Adeno‐NRIP‐GFP (lane 8). NRIP‐Flag produced by pAAV‐NRIP‐Flag plasmid transfection for 48 hours (lane 6) had the best expression detected by western blot.

**Figure S2.** Detection of anti‐NRIP autoantibody in 43 patients with MG. The protein lysates of GFP and NRIP‐Flag, produced by pAAV‐GFP‐Flag and pAAV‐NRIP‐Flag plasmid transfection, respectively, used as antigens in western blot; GFP was used as the negative control. The sera of 43 patients with MG, which were diluted at a ratio of 1:1000, were used as the primary antibodies. Sera showing positive signals with NRIP‐flag but negative with GFP are anti‐NRIP seropositive.

**Figure S3.** The epitope mapping of anti‐NRIP autoantibody from six patients with MG. The epitope against NRIP autoantibody is mainly on the C‐terminal but excluding WD6 and WD7 domains of NRIP.

**Figure S4.** Detection of anti‐NRIP autoantibody in 19 control subjects. (A) Sera showing positive signals with NRIP‐flag but negative with GFP are anti‐NRIP seropositive. Six controls have anti‐NRIP autoantibody (N1, N4, N7, N8, N11, and N16). (2) Ten control subjects received the second test for the detection of anti‐NRIP autoantibody with a mean interval of 5.0 ± 0.8 months. The results of the two analyses were consistent.

**Figure S5.** The epitope mapping of anti‐NRIP autoantibody from six healthy control subjects. The epitope against NRIP autoantibody is mainly on the C‐terminal but excluding WD6 and WD7 domains of NRIP.

**Figure S6.** IgG subclass analysis for anti‐NRIP autoantibody from six healthy control subjects. The western blot shows that all the six anti‐NRIP seropositive control subjects had anti‐NRIP autoantibodies belonging to IgG1, while one of them (N11) also had IgG3 subclass of anti‐NRIP autoantibody.
