# Supplementary figures and images for: Autoantibody of NRIP, a novel AChR‐interacting protein, plays a detrimental role in myasthenia gravis
Source: J Cachexia Sarcopenia Muscle. 2021 Mar 26;12(3):665–76. doi: 10.1002/jcsm.12697 (PMC8200423; doi:10.1002/jcsm.12697)

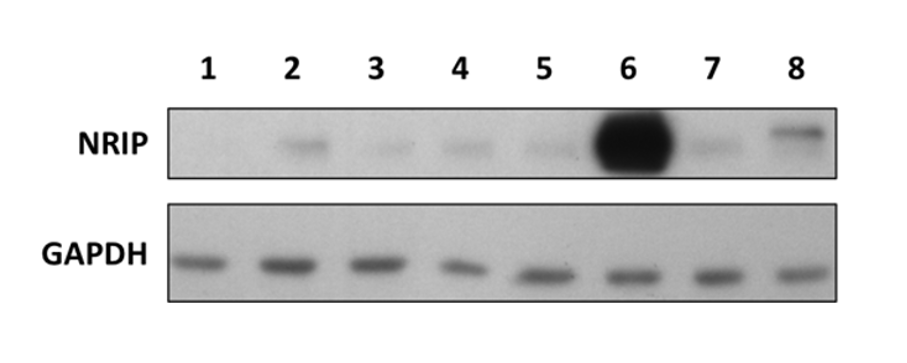

Supplement: Supplementary file 1 — Figure S1. NRIP protein was produced from HEK293T cells in different ways, including plasmid transfection with 3xFlag vector by jetPRIME (lane 1); 3xFlag‐NRIPby jetPRIME (lane 2), calcium phosphate with 5 μg 3xFlag‐NRIP (lane 3) or 10 μg 3xFlag‐NRIP (lane 4); with pAAV‐GFP plasmid DNA (lane 5) and pAAV‐NRIP‐Flag plasmid DNA (lane 6) by jetPRIME; and infection by AAV virus encoding NRIP‐Flag (lane 7) and plasmid DNA Adeno‐NRIP‐GFP (lane 8). NRIP‐Flag produced by pAAV‐NRIP‐Flag plasmid transfection for 48 hours (lane 6) had the best expression detected by western blot. [file JCSM-12-665-s003.tif]

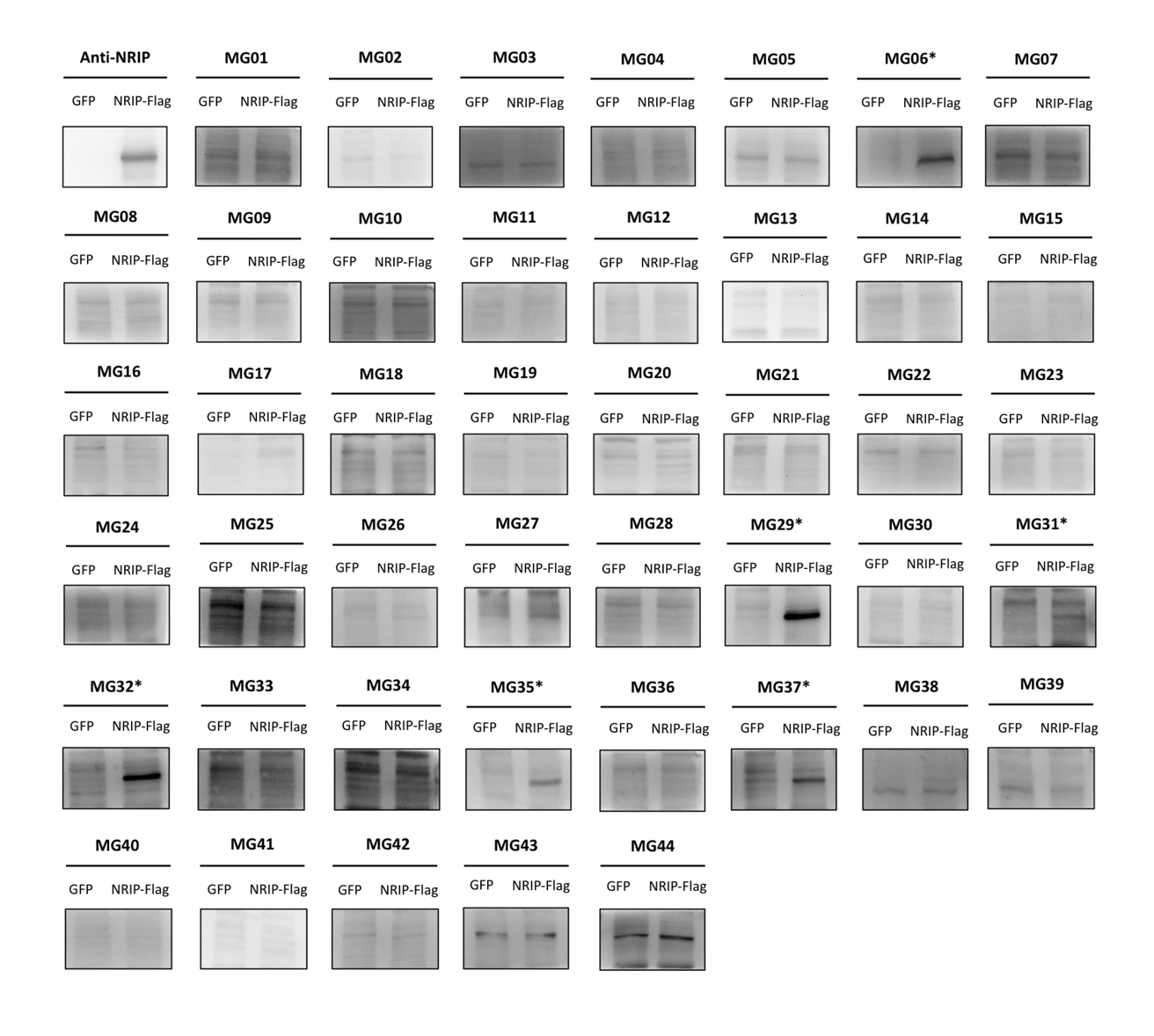

Supplement: Supplementary file 2 — Figure S2. Detection of anti‐NRIP autoantibody in 43 patients with MG. The protein lysates of GFP and NRIP‐Flag, produced by pAAV‐GFP‐Flag and pAAV‐NRIP‐Flag plasmid transfection, respectively, used as antigens in western blot; GFP was used as the negative control. The sera of 43 patients with MG, which were diluted at a ratio of 1:1000, were used as the primary antibodies. Sera showing positive signals with NRIP‐flag but negative with GFP are anti‐NRIP seropositive. [file JCSM-12-665-s006.tif]

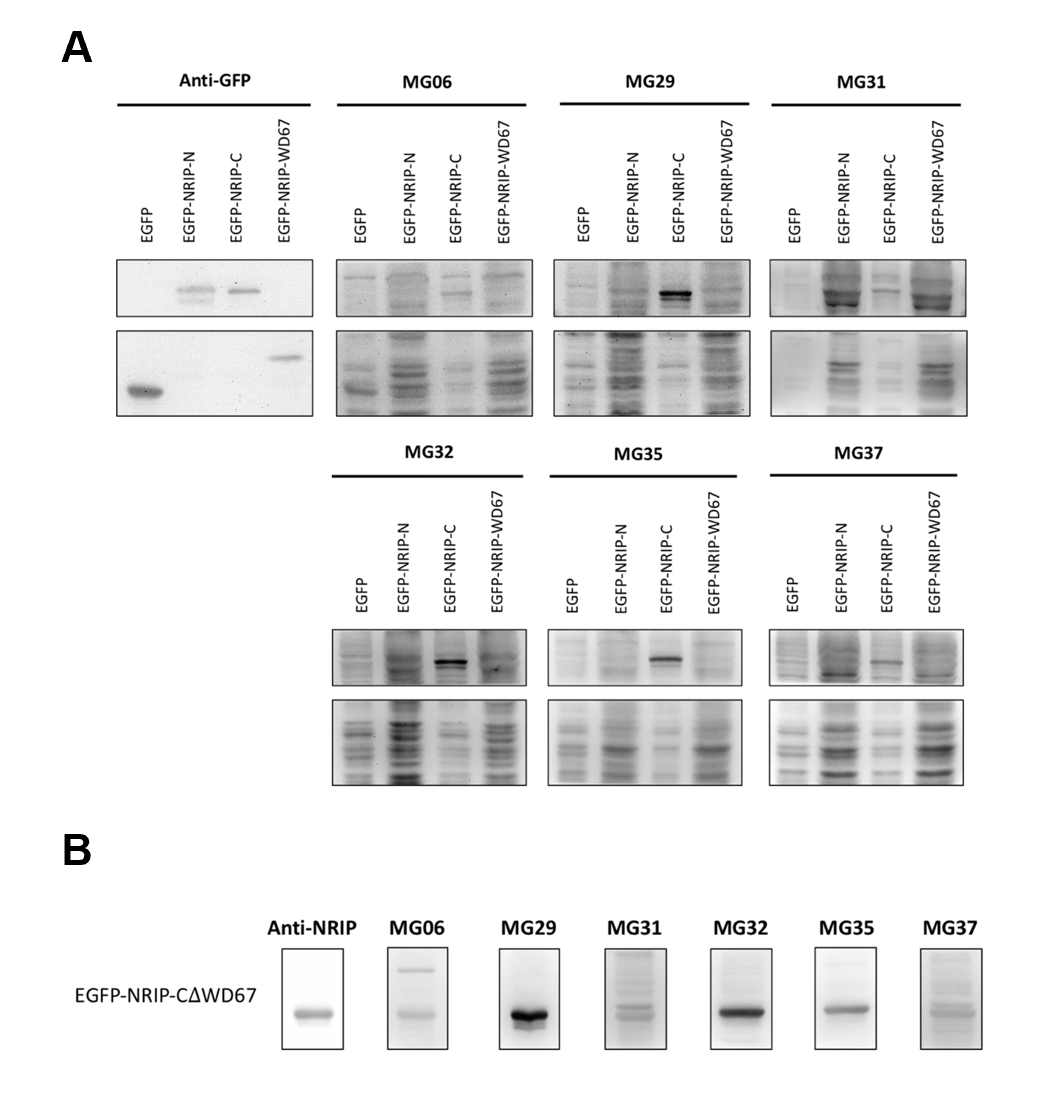

Supplement: Supplementary file 3 — Figure S3. The epitope mapping of anti‐NRIP autoantibody from six patients with MG. The epitope against NRIP autoantibody is mainly on the C‐terminal but excluding WD6 and WD7 domains of NRIP. [file JCSM-12-665-s004.tif]

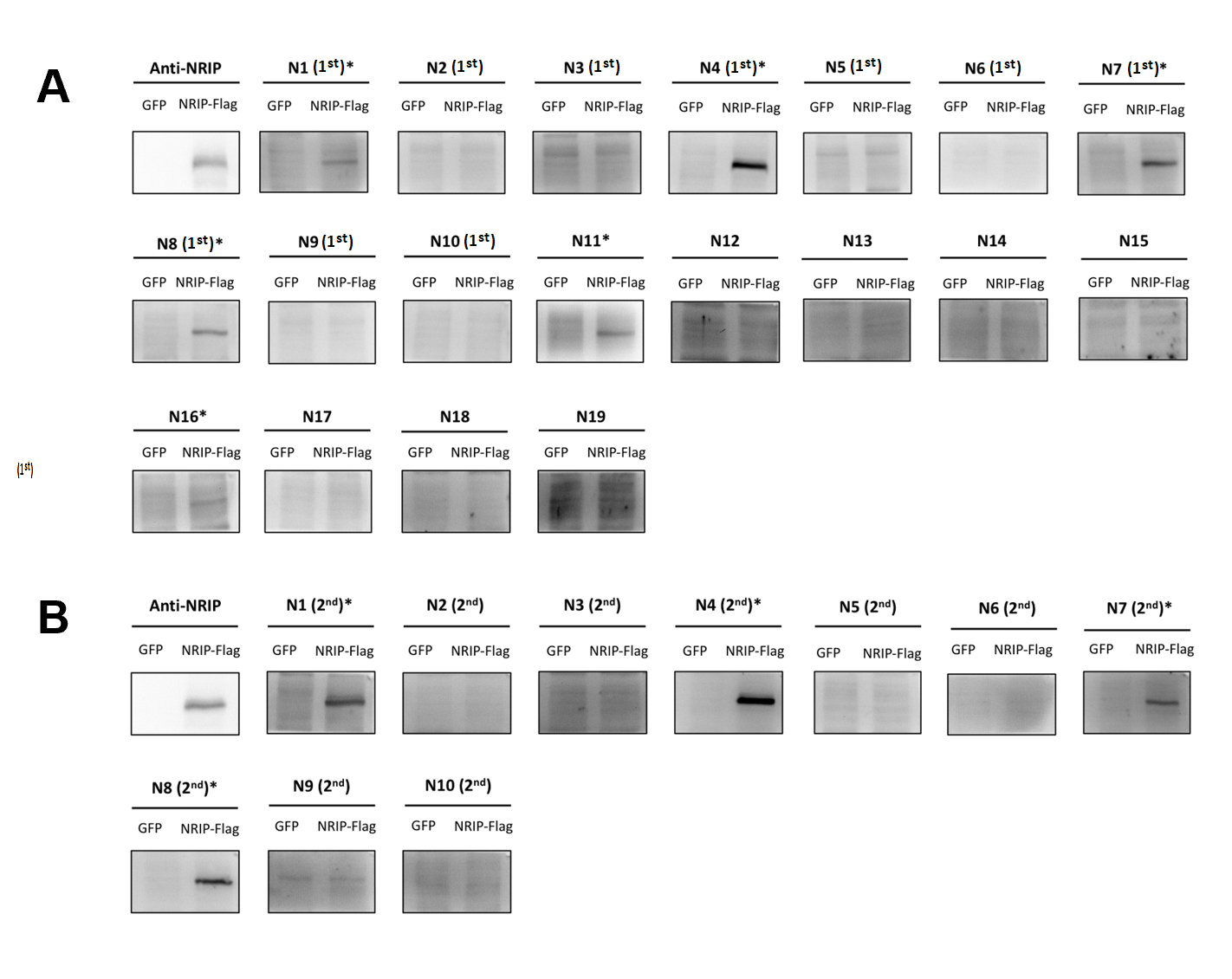

Supplement: Supplementary file 4 — Figure S4. Detection of anti‐NRIP autoantibody in 19 control subjects. (A) Sera showing positive signals with NRIP‐flag but negative with GFP are anti‐NRIP seropositive. Six controls have anti‐NRIP autoantibody (N1, N4, N7, N8, N11, and N16). (2) Ten control subjects received the second test for the detection of anti‐NRIP autoantibody with a mean interval of 5.0 ± 0.8 months. The results of the two analyses were consistent. [file JCSM-12-665-s001.tif]

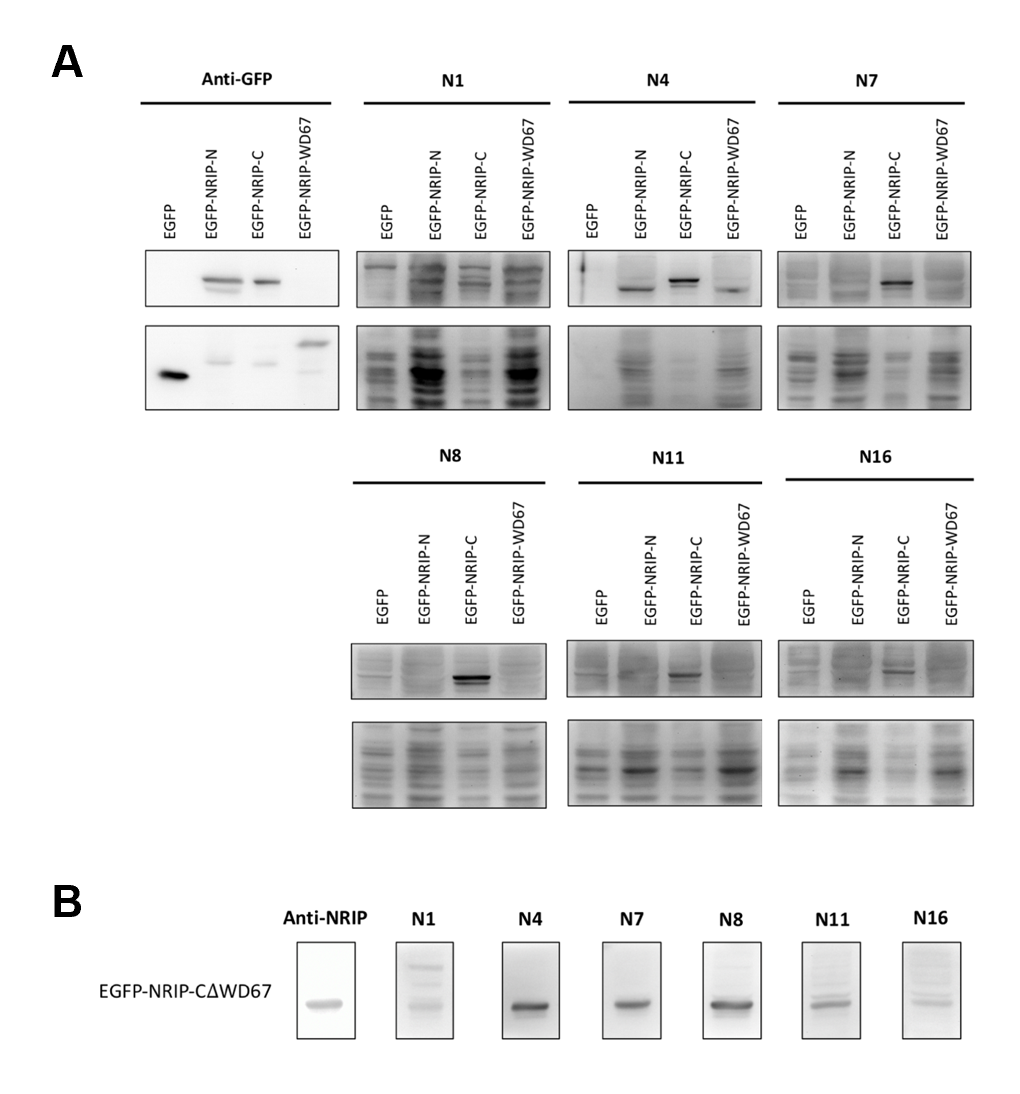

Supplement: Supplementary file 5 — Figure S5. The epitope mapping of anti‐NRIP autoantibody from six healthy control subjects. The epitope against NRIP autoantibody is mainly on the C‐terminal but excluding WD6 and WD7 domains of NRIP. [file JCSM-12-665-s005.tif]

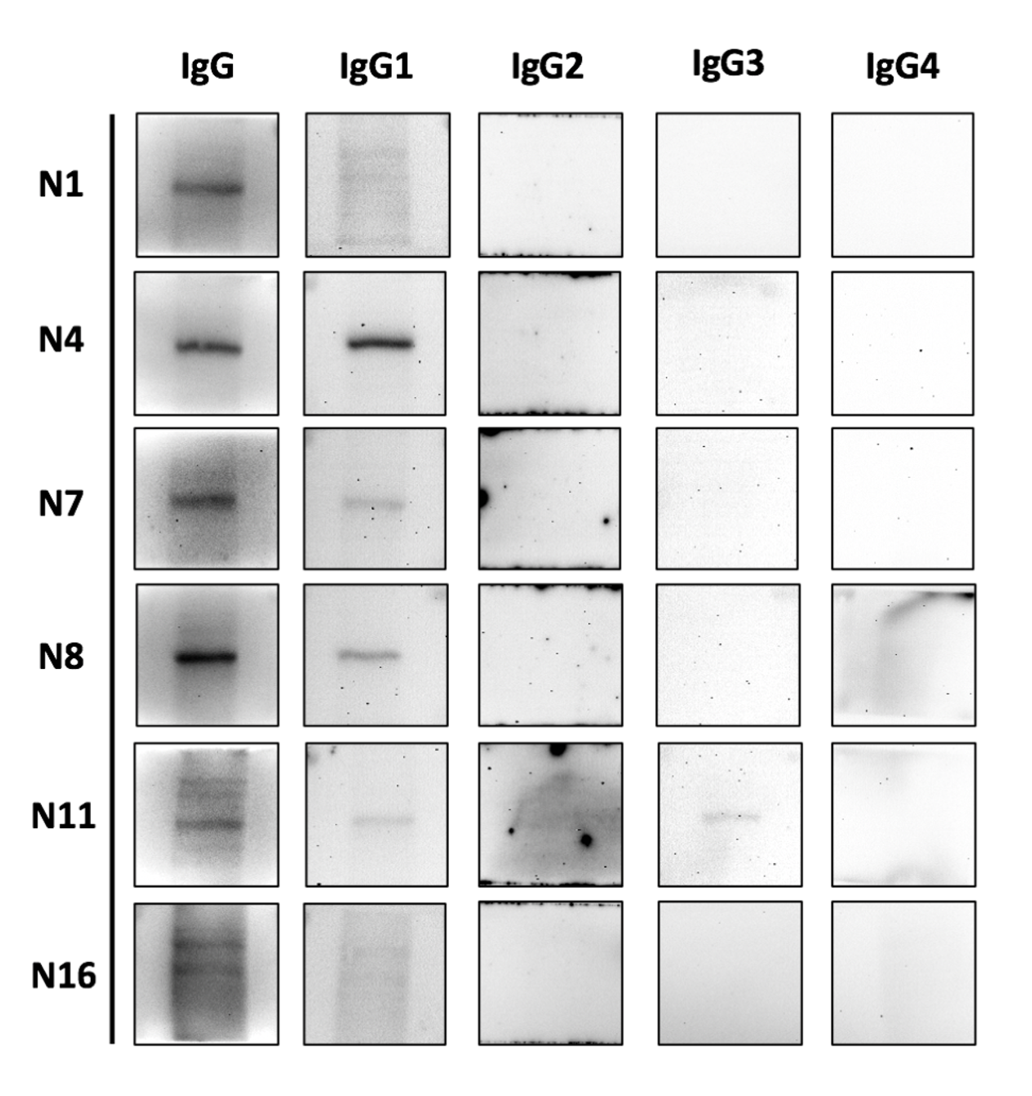

Supplement: Supplementary file 6 — Figure S6. IgG subclass analysis for anti‐NRIP autoantibody from six healthy control subjects. The western blot shows that all the six anti‐NRIP seropositive control subjects had anti‐NRIP autoantibodies belonging to IgG1, while one of them (N11) also had IgG3 subclass of anti‐NRIP autoantibody. [file JCSM-12-665-s002.tif]
